# The Effect of Applied Dance Therapy on Life Satisfaction and Fear of Happiness Among Turkish Board High School Students

**DOI:** 10.3390/healthcare13040392

**Published:** 2025-02-11

**Authors:** Neşe Genç, Zarife Taştan, Abdullah Demirli, Gülsüm Yılmaz, Burcu Güvendi, Selin Biçer Baikoğlu, Sevim Güllü, Cemre Can Akkaya

**Affiliations:** 1 Ministry of National Education, Ziyapaşa Middle School, Adana 01140, Turkey; 2 Faculty of Sport Sciences, Istanbul University Cerrahpaşa, Istanbul 34320, Turkey; zarife.tastan@iuc.edu.tr (Z.T.); selin.bicerbaikoglu@iuc.edu.tr (S.B.B.); sevim.gullu@iuc.edu.tr (S.G.);; 3 Faculty of Sport Sciences, İstanbul Topkapı University, Istanbul 34330, Turkey; gulsumyilmaz@topkapi.edu.tr; 4 Faculty of Sport Sciences, Yalova University, Istanbul 77200, Turkey; burcu.guvendi@yalova.edu.tr; 5 Faculty of Sport Sciences, Istanbul Yeni Yüzyıl University, Istanbul 34010, Turkey

**Keywords:** dancing, fear of happiness, life satisfaction, mental health, physical activity, Zumba

## Abstract

Background/Objectives: In this study, we aimed to examine the effect of a 12-week dance (Zumba) performance on the fear of happiness and life satisfaction perceptions of high school students studying at boarding school. Methods: The sample group of this study consisted of high school students staying in the school dormitory in the 2022–2023 academic year. A total of 82 students between the ages of 15 and 17, selected by the convenience sampling method, participated in the study, and 41 of the students were selected as the experimental group and 41 as the control group. The Fear of Happiness Scale and Satisfaction with Life Scale were used in the study. The measurement tools were administered as a pre-test one week before the dance program started and as a post-test one week after the end of the program. Two-way ANOVA for mixed measures was applied to determine whether the fear of happiness and life satisfaction pre-test scores were statistically significantly different between the experimental and control groups and to compare the pre-test and post-test difference scores. Results: As a result of the research, it was concluded that the fear of happiness of the students who participated in the dance activity decreased, and their life satisfaction levels increased. These findings emphasize that dance can be an important resource that can increase the psychological well-being of young people. Conclusions: As a result, it can be said that the 12-week dance activity positively affected students’ fear of happiness and life satisfaction perceptions.

## 1. Introduction

Nowadays, research on improving the mental health of adolescents and increasing their overall life satisfaction is gaining importance. The school environment is an important platform where students’ academic and social development is shaped while also providing an opportunity to address their mental and emotional needs. During adolescence, especially in closed social environments such as boarding schools, environments with limited emotional support mechanisms have a significant impact on the psychological well-being of individuals [1]. School-based intervention programs aimed at improving mental health have strategic significance, as they can directly impact students’ lives. School-based intervention programs are systematic programs implemented to support students’ academic, social, emotional, and behavioral development in the educational environment [2]. The development of such programs has become a necessity to meet the multifaceted needs of students, such as stress, peer relationship problems, academic pressures, and emotional difficulties [3]. These intervention programs, especially in school settings, aim to increase students’ psychological resilience, improve quality of life, and reduce negative emotional states at individual and group levels. School-based intervention programs are based on social–emotional learning (SEL) approaches. SEL provides a systematic framework for students to develop self-awareness, self-regulation, empathy, decision-making, and social skills. Research shows that SEL-based programs not only improve students’ academic performance but also positively affect their ability to cope with stress and emotional well-being [3].

In recent years, innovative approaches such as dance and movement-based programs have also been integrated into school-based interventions. Such approaches are supported by studies highlighting the positive effects of physical activity and dance on mental health [4,5]. It is known that physical activities and group social activities are effective in improving individuals’ life satisfaction and perceptions of happiness [6,7]. Studies in the literature indicate that dance has stress-reducing and self-confidence-enhancing effects on individuals [8,9]. Group movement activities such as dance allow individuals to express themselves and can also function as a form of social support through group dynamics [9,10].

Social and emotional changes, especially during adolescence, may cause individuals to develop negative perceptions of happiness. Fear of happiness is defined as individuals’ anxiety about the continuation of negative outcomes for various reasons during the process of feeling positive emotions and experiencing happiness [11]. Fear of happiness may affect individuals’ motivation to desire and maintain happiness in the long term [12]. Studies in the literature show that physical activity contributes to a reduction in fear of happiness by creating positive effects on happiness perception and life satisfaction [13]. In particular, dance activities can support this process by allowing individuals to establish healthier relationships with their social environment and express their emotions freely. In addition, research indicates that dance increases individuals’ physical awareness and contributes positively to the perception of happiness [8]. It has been stated that individuals who think that their lives are within satisfactory limits have fewer negative emotions, are generally satisfied with their lives, and have an increased sense of happiness [14]. The state of satisfaction that emerges when life is considered holistically is indicated by the concept of life satisfaction [15]. It is known that the level of life satisfaction in adolescents has a direct impact on their emotional health and interactions with their social environment [16]. There are many studies suggesting that dance increases life satisfaction by strengthening individuals’ body perception and self-confidence [4,7]; energetic and fun dance types, especially Zumba, contribute to psychological well-being by increasing individuals’ sense of belonging within a group [17], while the fact that dance is a multifaceted artistic practice involving sensory, motor, cognitive, social, emotional, rhythmic, and creative processes makes it different from other physical activities [18]. Unorthodox forms of physical activity such as aerobics, Zumba dances, and cultural dances are preferred because they are fun, exciting, and psychologically positive. It is thought that these activities can support psychological well-being in a multidimensional way by organizing different types of dances in accordance with individuals’ ages, personal interests, and socio-cultural environments [9,11]. Considering that they have physical and mental health effects [19], these activities can significantly impact students distancing themselves due to a fear of happiness and life satisfaction. Dance-based intervention programs contribute to students’ psychological and social well-being by combining social–emotional learning approaches with physical activity. In this context, dance activities are considered an area of interest among school-based intervention programs [8]. Creative activities such as dance and music can strengthen the impact of intervention programs by increasing students’ motivation.

Along with negative effects such as anxiety and stress caused by the fear of happiness in boarding high school students, it is important that dance (Zumba) performance makes them feel psychologically positive emotions and the feeling of having a healthy body that comes with physical activity in their perception of life satisfaction. When the literature is examined, it is seen that there are studies examining the fear of happiness and life satisfaction perception using different physical activity methods and various sample groups of students staying in boarding high school dormitories away from their families [20,21,22,23]. However, studies in the literature that explore the impact of Zumba dance exercises on fear of happiness and life satisfaction perception—while including both a fun physical activity like Zumba and looking at students staying in boarding schools away from their families—are limited. This situation makes the originality of this study and its contribution to the literature even more important. This study is based on the hypothesis that a 12-week Zumba dance program will reduce the fear of happiness and positively increase the life satisfaction perceptions of high school boarding students. According to the findings of this study, it is expected to conclude that dance programs can be used to increase students’ psychological well-being and that such physical activity programs can be evaluated as a tool to reduce negative emotional states such as fear of happiness and that dance exercises can be recommended to increase their life satisfaction.

## 2. Method

### 2.1. Research Design

This study was designed in a quasi-experimental design with a pre-test–post-test control group in which paired groups were randomly assigned as experimental groups. The design allowed the researcher to compare the pre-test scores of the experimental and control groups before the experimental manipulation [24].

### 2.2. Participants

The sample of this study consists of students who received boarding education in high school dormitories in the 2022–2023 academic year. Students from the 9th, 10th, and 11th grades were selected for the study. The 12th-grade students were not included as they were preparing for their university entrance exam. Only boarding school students were included in the study. After the COVID-19 pandemic, it is believed that adolescents have experienced an increase in mental health issues, with students in boarding schools being more affected by negative impacts such as social isolation, anxiety, and stress. In this context, students from boarding schools were included in this study. The sample group consisted of 82 female students ($N_{control}$ = 41, $N_{experiment}$ = 41) selected by purposive sampling, specifically criterion sampling. In Turkish culture, there is a perception that Zumba dance is primarily a dance performed by women, which is why only a few male students volunteered for the study. As a result, male participants were excluded from the study. A sample pool consisting of students with similar happiness, life satisfaction scores, and other socio-demographic characteristics was created, taking into account the criteria set for including the sample group in the study. The students were randomly assigned to the experimental and control groups from this sample pool. With random assignment, the aim was to form two equal groups (experimental and control) for the feature to be measured. The sample number of the study was determined to be 82, with 41 participants in each of the groups ($N_{control}$ = 41, $N_{experiment}$ = 41), calculated in the G-Power 3.1.9.7 program with a moderate effect size, a margin of error of 0.05 and a test power of 0.80, as recommended by ref [25] for studies in the field of social sciences; the dropout status of the participants was also considered in this study. The inclusion criteria were determined as being a female student, receiving boarding education, being a volunteer, having their family’s consent to participate in the study, and having no previous dance experience. The exclusion criteria were as follows: being a 12th-grade student preparing for the exam, having serious health issues such as heart disease, severe asthma, back, and knee problems, having serious orthopedic issues that impair mobility, having psychological disorders that would prevent participation in group activities like Zumba (such as severe depression and severe anxiety disorders), and having mental or developmental disabilities that would prevent benefiting adequately from the physical and social benefits of the activity, including receiving psychological support, and not participating regularly in the study. Additionally, students who participated in extracurricular activities were excluded from the study in order to control external factors.

### 2.3. Data Collection Tools

In this study, the Fear of Happiness Scale and Satisfaction with Life Scale were used as data collection tools. Zumba, a popular dance among adolescents, is assumed to have the potential to improve mental health due to its enjoyable nature and physical activity. Therefore, Zumba was chosen as the intervention method for this study. The scales used in the study were administered to the students randomly assigned to the experimental and control groups before the dance activities started. The students in the experimental group participated in the dance activity 2 days a week for 12 weeks, while the control group students did not participate in this activity. At the end of 12 weeks, the research scales (post-test) were applied to both the control and experimental groups for the second time. A total of 12 weeks is generally considered a sufficient period to observe the effects of a program. This duration allows participants to adapt to the program and enables the observation of meaningful changes in their behaviors or emotional/psychological states.

#### 2.3.1. School-Based Intervention Program: Zumba Activity

##### Purpose of Program Development

This program aims to promote the social, emotional, and psychological development of young people, enhance their physical health, and improve educational and social skills. Zumba was chosen as an enjoyable activity to increase happiness and life satisfaction.

##### Reason for Program Development

Adolescence is a period of rapid emotional and psychological growth, with increased stress and anxiety. Female students in boarding schools may face challenges related to social connections and psychological support. Zumba is chosen to improve physical health, boost happiness through endorphins, and help students manage stress, fostering healthier lifestyles and emotional balance.

##### Program Goals

Physical Health and Psychosocial Development: Zumba enhances physical health and body awareness, contributing to self-confidence, social skills, and life satisfaction.

Supporting Psychological Well-being: Zumba helps students manage stress and psychological issues such as anxiety and depression. Measuring happiness and life satisfaction post-program is key to understanding its psychological impact.

Social Interaction and Community Bonds: Zumba encourages group interactions, strengthens social bonds, and develops positive social skills.

##### Program Implementation Method

The program ran for 12 weeks with Zumba activities held twice a week. Students participated in dynamic, enjoyable sessions that improved their physical health while learning the correct dance movements and enjoying group engagement.

##### Program Evaluation

The program’s effectiveness was assessed using happiness, fear, and life satisfaction scales before and after the program. Changes in these factors helped evaluate the program’s impact.

#### 2.3.2. Fear of Happiness Scale

The Fear of Happiness Scale developed by Joshanloo in [23] was adapted to the Turkish culture by ref [26]. The scale consists of 5 items, and there are no items that need to be reverse-coded. The scale is a 7-point Likert-type scale that is evaluated between “Strongly Disagree” and “Strongly Agree”. The higher the scores obtained from the scale, the higher the fear of happiness. In our study, the internal consistency reliability coefficient was calculated as 0.72. Confirmatory factor analysis (CFA) was applied to check whether the scale provides model-data fit in adolescents. When the fit indices were checked, it was determined that the model and data were compatible. Therefore, the scale can be used in adolescents (NFI = 0.96, RMSEA = 0.08, CFI = 0.97, GFI = 0.92, AGFI = 0.89 and SRMR = 0.04). The items of the scale are provided below:

“I prefer not to be too joyful because I believe that sorrow follows joy.”
“I believe that the more cheerful and happy I am, the more prepared I should be for bad things to happen in my life.”
“After good luck or fortune, disasters usually follow.”
“Too much joy/pleasure and fun invite bad things to happen.”
“Excessive joy has some negative consequences.”

#### 2.3.3. Satisfaction with Life Scale

The Satisfaction with Life Scale developed by [27] was adapted to Turkish culture by [28]. The scale consists of 5 items and shows a single-factor structure. A low score on this scale is accepted as an indicator of low life satisfaction. Students evaluated the 5 items with a 5-point Likert-type rating between “strongly disagree” and “strongly agree”. In our study, Cronbach’s Alpha internal consistency coefficient was 0.87. Confirmatory factor analysis (CFA) was applied to check whether the scale provides model-data fit in adolescents. When the fit indices were checked, it was determined that the model and data were compatible. Therefore, the scale can be used in adolescents (NFI = 0.89, RMSEA = 0.08, CFI = 0.90, GFI = 0.89, AGFI = 0.85 and SRMR = 0.09). The items of the scale are provided below:

“I have a life that is close to my ideals.”
“My living conditions are perfect.”
“I am satisfied with my life.”
“So far, I have achieved the important things I wanted from life.”
“If I were to be born again, I would change almost nothing in my life.”

### 2.4. Collection of Data

In this study, a “pre-test–post-test control group design”, as an experimental design, was used to examine the effect of dance programs on high school students’ fear of happiness and life satisfaction. Ethics committee approval is dated 24 February 2022 and numbered 2022/03-14 and was obtained from Istanbul Esenyurt University Ethics Committee for the implementation of the research. Voluntary consent forms were obtained from the students participating in the study and their parents. Forty-one female students were selected for each of the experimental and control groups. The age group of the students and the duration of their boarding at the school were taken as criteria for sample selection. The selected students were informed about the study and information, and consent forms were obtained from the students. The pre-test measurements of the measurement tools used in the study were administered one week before the program started. The students in the experimental group participated in the dance activity for 40 min 2 days a week for 12 weeks, while the control group students did not participate in this activity. At the end of 12 weeks, the scales used in the study were applied to both the control and experimental groups for the second time as part of the post-test 1 week after the program ended.

#### 2.4.1. Dance Program

Zumba aims to improve physical health and support students’ psychological and social development.

Physical Goals: Improve cardiovascular endurance, strengthen muscles, and enhance coordination. This helps to establish regular physical activity habits.

Psychological Goals: Help students cope with stress, reduce anxiety and depression, and increase happiness. This fosters self-confidence and self-esteem.

Social Goals: Encourage group interaction and social skill development and emphasize the power of community and collective movement.

##### Implementation Procedure

Duration and Frequency: Zumba sessions were held twice a week for 12 weeks, totaling 24 sessions, each lasting 40 min.

Instructor Selection: A skilled instructor led the sessions, providing appropriate movements and guiding group dynamics.

Warm-up and Cool-down: Each session included 5–10 min of warm-up and cool-down exercises to prevent injuries and relax the body.

Customized Movements: Dance styles ranged from Latin rhythms to popular music, with lessons tailored to students’ age and skill levels.

Feedback and Support: The instructor provided continuous feedback, encouraged proper movement, and fostered a supportive group environment. The Zumba dance program that was applied is presented in Table 1.

### 2.5. Analysis of Data

Before the data analysis, missing data were analyzed, outliers were identified, and the normality assumption of the data was tested. Skewness and kurtosis values were calculated to determine whether the data met the normality assumption. Since the skewness and kurtosis values were between −2 and +2, it was assumed that the variables were normally distributed [29]. Then, confirmatory factor analyses were conducted to check whether the scales used in the study provided model data fit in adolescents. Two-way ANOVA for mixed measures was applied to determine whether the fear of happiness and life satisfaction pre-test scores had statistically significant differences between the experimental and control groups and to compare the pre-test and post-test difference scores. The SPSS 25.0 program was used for descriptive statistics of the data, and two-way ANOVA was used for mixed measures. The LISREL 8.7 program was used in the confirmatory factor analysis of the scales.

### 2.6. Findings

Descriptive statistics for the pre-test and post-test fear of happiness scores of the experimental and control groups are presented in Table 2.

As shown in Table 2, the pre-test mean of fear of happiness in the experimental group was 4.94 ± 0.86, and the post-test mean was 3.72 ± 0.95; the pre-test mean of fear of happiness in the control group was 5.20 ± 0.96, and the post-test mean was 5.21 ± 0.90. When the descriptive statistics are analyzed, it can be seen that the mean scores of the pre-test are close to each other in the experimental and control groups. After the dance program was applied to the experimental group, it can be seen that the average fear of happiness in the experimental group decreased significantly and increased very slightly in the control group. In order to determine the significance of the difference between these averages, two-way ANOVA results for mixed measures are given below.

When Table 3 is examined, a significant difference can be observed in the fear of happiness levels of the students in the experimental and control groups before and after the experiment (F(1, 80) = 59.52, *p* < 0.05, $\eta^{2}$ = 0.43). In other words, the joint effects of being in different treatment groups and the repeated measures that are factors of fear of happiness were found to be significant. When the eta squared value is analyzed, 0.43 indicates that the effectiveness of dance on fear of happiness is at a high level.

On the other hand, when the main effect of the measurement factor is analyzed regardless of the group variable, it can be said that the differentiation between the test scores of the individuals is significant (F(1, 80) = 57.64, *p* < 0.05, $\eta^{2}$ = 0.42). In this case, dance was effective at reducing the level of fear of happiness. When the effect of the group variable on the level of fear of happiness without considering the measurement factor was examined, a significant difference was observed (F(1, 80) = 21.88, *p* < 0.05, $\eta^{2}$ = 0.22). In other words, it can be said that the group variable alone is an effective factor in students’ fear of happiness levels. Descriptive statistics for the pre-test and post-test satisfaction with life scores of the experimental and control groups are presented in Table 4.

As shown in Table 4, the pre-test mean of life satisfaction for the experimental group was 3.38 ± 0.70, and the post-test mean was 3.93 ± 0.54; the pre-test mean of life satisfaction for the control group was 3.47 ± 0.87, and the post-test mean was 3.66 ± 0.74. When the descriptive statistics are analyzed, it can be seen that the mean scores of the pre-test are close to each other in the experimental and control groups. After the dance program was applied to the experimental group, it can be seen that the mean life satisfaction of the experimental group increased significantly, while it increased slightly in the control group. In order to determine the significance of the difference between these averages, two-way ANOVA results for mixed measurements are given below.

When Table 5 is examined, a significant difference can be observed in the life satisfaction levels of the students in the experimental and control groups before and after the experiment (F(1, 80) = 13.05, *p* < 0.05, $\eta^{2}$ = 0.14). In other words, the joint effects of being in different treatment groups and the repeated measures of factors of life satisfaction were found to be significant. When the eta squared value is analyzed, 0.14 shows that the effectiveness of the dance on life satisfaction is at a moderate level.

On the other hand, when the main effect of the measurement factor is analyzed regardless of the group variable, it can be said that the differentiation between the test scores of the individuals is significant (F(1, 80) = 57.31, *p* < 0.05, $\eta^{2}$ = 0.42). In this case, dance was effective at increasing the level of life satisfaction. When the effect of the group variable on the level of life satisfaction without considering the measurement factor is examined, it can be seen that there is no significant difference (F(1, 80) = 0.31, *p* > 0.05). In other words, it can be said that the group variable alone is not an effective factor in students’ life satisfaction levels.

## 3. Discussion and Conclusions

This study contributes to the literature in this field by examining the effects of a dance-based intervention program to support mental health in the school environment. The aim of this study was to examine the effect of participation in dance (Zumba) activities on the fear of happiness and life satisfaction perceptions of high school students attending boarding schools. The COVID-19 pandemic and subsequent global uncertainties, physical health concerns, AND disruptions in education and daily life have led to increased feelings of loneliness, decreased mental well-being, and increased fear of happiness in the adolescent population worldwide [30,31,32]. Moreover, the increase in various psychological problems, especially depression and sleep disorders in the short term and pandemic-related post-traumatic stress disorder in the long term, has reached an alarming level [33].

Schools not only encourage academic success but also play an important role in protecting and improving the mental health of young people [33]. Integrating dance and movement-based activities into school-based intervention programs has the potential to have far-reaching effects in terms of improving and supporting students’ mental health. One of the key elements of mental health promotion is strengthening social connections between students. Dance-based activities, which are usually carried out in the form of group activities, play an important role in ensuring social cohesion and reducing feelings of loneliness, especially for students studying in boarding schools [34]. In this context, the regular participation of high school students staying in boarding school dormitories in Zumba activities is expected to contribute to a reduction in their psychological problems and positive changes in their behaviors in the long term. It is observed that scientific studies on Zumba in schools are extremely limited, and physical fitness and health dimensions are mostly investigated.

As a result of this research, there was a statistically positive change in the fear of happiness scores in favor of experimental group students who participated in dance (Zumba) activities. It was found that the fear of happiness of the students participating in dance activities decreased, while it increased very slightly in the control group. Dance draws attention as a physical activity that can improve individuals physically [35] as well as impact psychosocial factors such as cognitive ability, sense of community, success, and vitality [5]. In the literature, there are studies [4,36,37] that reveal the positive effects of dance on individuals as a result of its evaluation of many psychological parameters. While dance has positive effects on human psychology even when performed individually, when performed systematically, and with other participants, it plays an important role in promoting socialization [10,38] as well as reducing or completely eliminating mental and nervous tension [39,40].

People spontaneously and unconsciously synchronize with the music while dancing, and this is known to improve both mood and the ability to withstand strenuous exercise [34]. The concept of fear of happiness is also considered by some psychiatrists as a type of anxiety disorder [41]. Psychological illnesses, one of the diseases of our age, can be treated with “Dance Therapy” today. In ref [39], a study was conducted aiming to evaluate the effectiveness of dance therapy in reducing stress and anxiety; it was emphasized that dance therapy provides significant positive effects in reducing anxiety and stress levels. In a 2019 study, it was concluded that dance therapy is useful in the processes of psychological counseling [40]. Gilbert et al. [20] found a significant relationship between fear of happiness and depression, anxiety, and stress. The findings of their study revealed that fear of happiness can have negative effects on the psychological state of individuals and is frequently associated with psychological disorders such as depression, anxiety, and stress. In a study conducted in ref [41], it was found that young people’s participation in dance activities led to a significant reduction in depression and anxiety levels and also provided many positive psychological and physiological contributions. These findings emphasize the psychological benefits of dance on young people and reveal the positive effects of physical activity, especially on mental health. These studies are in agreement with our study in this respect.

Through dance, individuals escape from daily issues and feel happy [42]. In Ceylan and Kozak’s [43] study, it was found that the concept of Zumba is associated with metaphors that contribute to mental health, such as distancing from negative emotions, happiness, and feeling good through entertainment and dance. In the study, the metaphors expressed in relation to Zumba were classified under three main categories: “psychological benefit”, “feeling of happiness” and “pleasure”. It is known that young people in high school have complex moods brought on by adolescence, which is a stormy period, and that they experience individual conflicts, indecisions, and concerns about the future [44]. In this period, Zumba activities are thought to positively affect their perception of fear of happiness.

Another finding of this study is that there was a statistically significant positive change in life satisfaction scores for the experimental group of students who participated in dance activities. Students in the control group showed a very low level of increase. In the study conducted by Tunçgenç et al. [38], it was found that a 5-week group dance program strengthened the social bonds of adolescents, and this development showed a positive relationship with increased life satisfaction and a positive future orientation. In Cabbaroğlu’s [45] study examining the effects of Zumba and Pilates activities on life satisfaction and happiness, it was determined that the frequency of women’s participation in Zumba and Pilates activities significantly increased their life satisfaction levels. Han and Sa’s [46] study showed that participation in dance activities had a positive and significant impact not only on individuals’ physical health but also on their daily life satisfaction. This study reveals that the participants not only maintained their physical health through dance but also felt a higher level of satisfaction with their daily lives, emphasizing that dance can be considered a multifaceted activity that increases the quality of life and overall happiness. The findings of this study support the findings of our study, which show similar characteristics.

The research clearly demonstrates the potential of integrating dance-based interventions into school-based programs to support mental health. Adolescence, in particular, is a critical period in which students cope with emotional and social challenges and develop stress-coping skills. In this period, school-based interventions are needed to help students overcome psychological difficulties such as fear of happiness. In this study, it was observed that the 12-week Zumba program reduced this negative situation and increased students’ life satisfaction. The current findings suggest that dance-based creative interventions in the school environment can be an effective tool to support students’ mental health.

Moreover, this research highlights students’ interest in dance activities, particularly Zumba, and the positive energy they can derive from such activities, demonstrating the potential for incorporating movement-based practices more effectively into educational processes. To enhance students’ motivation and support their mental and physical health, Zumba could be introduced as an elective course at the primary, secondary, and tertiary education levels. This proposal aligns with the emphasis by Silva et al. [47] on the need to restructure full-time school curricula to prioritize physical activities within the school environment. Movement-based physical activities such as Zumba hold significant potential to enhance students’ physical and psychological well-being and could be integrated into regularly implemented psycho-social support programs in schools to support mental health. Adapting group-based dance activities to suit different educational levels (primary, secondary, and tertiary) through tailored models may further amplify their effectiveness. For example, game-based Zumba programs could be designed for primary school students, while stress management and group dynamics-focused programs could be tailored to high school students. Such customized approaches would make these activities more suitable for the developmental needs of various age groups.

Integrating dance-based activities into school-based interventions offers a number of advantages. First, dance programs do not require specialized equipment or extensive infrastructure, making them a low-cost and easy-to-implement intervention tool [48]. This increases the accessibility of dance-based interventions even for schools with limited economic resources. Moreover, the fun and inclusive nature of dance encourages the active participation of a wide range of students in the program. In addition, dance-based interventions allow for the development of positive habits in students, and these habits can create long-term behavioral changes that can support their future well-being [34]. In this context, dance programs are not only a short-term intervention tool but also contribute to the development of skills with lifelong benefits.

The inclusion of only female students and high school-level participants in this study could restrict the direct generalizability of the findings to other age groups, genders, or educational levels. Additionally, potential external factors such as social interactions and the overall atmosphere of the school environment could not be controlled during the implementation process. This suggests that the potential environmental and social factors contributing to the effects of dance exercises might have been overlooked in this study’s findings. Moreover, participants might have experienced improvements in their life satisfaction or perceptions of happiness not only due to the dance activity itself but also because of the sense of being part of an innovative and engaging program. Such effects could have introduced limitations when evaluating the unique impacts of the intervention program. The impact of dance on high school students (in terms of fear of happiness and life satisfaction) may be less pronounced compared to older individuals. This is because adolescents, particularly during high school years, might be more resistant to positive influences due to factors such as future anxiety and pessimism.

Future studies designed with a broader range of participants, including different age groups, genders, and risk categories, may enhance the generalizability of these findings. Additionally, adopting a more comprehensive control group design and systematically monitoring social interactions during the intervention process are recommended to better assess the influence of external factors. Such methodological approaches could provide a deeper understanding of the findings and allow for more robust conclusions.

## Figures and Tables

**Table 1:** Zumba dance program applied.

Week	Warm-Up (5 min)	Main Section (30 min)	Cool-Down (5 min)
1	Light cardio (brisk walking, stretching)	Basic steps: Merengue, Salsa (10 min each) + Free practice (10 min)	Light stretching, deep breathing
2	Basic stretching movements	Merengue and Salsa repetitions + simple routine practice	Dynamic stretching and relaxation
3	Light cardio and stretching	Introduction to Cumbia steps + repetition of previous steps	Breathing control and muscle relaxation
4	Basic dance movements	Cumbia and Reggaeton basic steps + simple combination	Light stretching and dance relaxation
5	Light cardio	Repetition of all basic steps (Merengue, Salsa, Cumbia, Reggaeton)	Deep breathing and muscle relaxation
6	Dynamic stretching	Coordinating arm and leg movements with dance + routine practice	Relaxation with light movements
7	Rhythmic cardio	Adding new moves (hip roll, pivot) + short choreography practice	Cool down with light dance movements
8	Rhythmic light warm-up	Repetition of all steps + full choreography attempt	Deep breathing and stretching
9	Basic cardio	Creating a medium-difficulty choreography and group work	Dynamic cool down and relaxation
10	Stretching and rhythm exercises	Enhancing choreography and stage practice	Relaxation with light movements
11	Light dance and warm-up	Full choreography practice and energy-boosting movements	Relaxation with dance
12	Motivational dance movements	Performance rehearsal and group showcase	Participant-chosen relaxation

**Table 2:** Descriptive statistics of pre-test and post-test fear of happiness scores of students in experimental and control groups.

Group	Test Period	n	$\bar{x}$	sd
Experimental group	Pre-test	41	4.94	0.86
	Post-test	41	3.72	0.95
Control group	Pre-test	41	5.20	0.96
	Post-test	41	5.21	0.90

**Table 3:** Two-way ANOVA results for mixed measures regarding the difference in fear of happiness scores between the experimental and control groups.

Type of Variance	Sum of Squares	df	Mean Squares	F	*p*	Eta Square
Group (experimental/control)	31.79	1	31.79	21.88	0.00	0.22
Measurement (pre-test/post-test)	14.88	1	14.88	57.64	0.00	0.42
GroupMeasurement	15.37	1	15.37	59.52	0.00	0.43
Error	20.65	80	0.26			
Total	178.27	163				

**Table 4:** Descriptive statistics of the pre-test and post-test satisfaction with life scores of the students in the experimental and control groups.

Group	Test Period	n	$\bar{x}$	sd
Experimental group	Pre-test	41	3.38	0.70
	Post-test	41	3.93	0.54
Control group	Pre-test	41	3.47	0.87
	Post-test	41	3.66	0.74

**Table 5:** Two-way ANOVA results for mixed measures regarding the difference in life satisfaction scores of the experimental and control groups.

Type of Variance	Sum of Squares	df	Mean Squares	F	*p*	Eta Square
Group (experimental/control)	0.30	1	0.30	0.31	0.579	0.004
Measurement (pre-test/post-test)	5.71	1	5.71	57.31	0.000	0.42
Group × Measurement	1.30	1	1.30	13.05	0.001	0.14
Error	7.97	80	0.10			
Total	92.69	163				

## Data Availability

Data is contained within the article.

## References

[1] Andersen S.L., Teicher M.H. (2008). Stress, sensitive periods and maturational events in adolescent depression. Trends Neuro Sci..

[2] Ayyıldız T. (2016). The analysis of the metaforic perceptions towards dance concept of university students. Gaziantep Univ. J. Sports Sci..

[3] Bailey R. (2006). Physical education and sport in schools: A review of benefits and outcomes. J. Sch. Health.

[4] Basso J.C., Satyal M.K., Rugh R. (2021). Dance on the brain: Enhancing intra-and inter-brain synchrony. Front. Hum. Neurosci..

[5] Burton L., Stinson S.W. (2016). The role of dance in promoting social-emotional learning. J. Dance. Educ..

[6] Büyüköztürk Ş., Çakmak E., Kılıç A., Özcan E., Karadeniz Ş., Demirel F. (2011). Scientific Research Methods.

[7] Cabbaroğlu M. (2019). Effect of Zumba and Pilates on Life Satisfaction and Happiness as Sportive Recreation Activity (Muğla Province Example). Master’s Thesis.

[8] Carey R.L., Bailey M.J., Polanco C.I. (2023). How the COVID-19 pandemic shaped adolescents’ future orientations: Insights from a global scoping review. Curr. Opin. Psychol..

[9] Ceylan G., Kozak M. (2021). Perceptions of active zumba members regarding the concept of “zumba”: A metaphor analysis study. Int. J. Hum. Sci..

[10] Cohen L., Manion L., Morrison K. (2018). Research Methods in Education.

[11] Cugusi L., Manca A., Bergamin M., Blasio A.D., Yeo T.J., Crisafulli A., Mercuro G. (2019). Zumba fitness and women’s cardiovascular health: A systematıc review. J. Cardiopulm. Rehabil. Prev..

[12] Dağlı A., Baysal N. (2016). Adaptatıon of the satısfactıon wıth lıfe scale ınto turkısh: The study of valıdıty and relıabılıty. Electron. J. Soc. Sci..

[13] Delextrat A.A., Warner S., Graham S., Neupert E. (2016). An 8-week exercise intervention based on Zumba improves aerobic fitness and psychological well-being in healthy women. J. Phys. Act. Health.

[14] Demirci İ., Ekşi H., Kardaş S., Dinçer D. (2016). The Validity and Reliability of Turkish Version of Fear of Happiness Scale. Kastamonu Educ. J..

[15] Diener E.D., Emmons R.A., Larsen R.J., Griffin S. (1985). The satisfaction with life scale. J. Personal..

[16] Durlak J.A., Weissberg R.P., Dymnicki A.B., Taylor R.D., Schellinger K.B. (2011). The impact of enhancing students’ social and emotional learning: A meta-analysis of school-based universal interventions. Child Dev..

[17] Fegert J.M., Vitiello B., Plener P.L., Clemens V. (2020). Challenges and burden of the Coronavirus 2019 (COVID-19) pandemic for child and adolescent mental health: A narrative review to high light clinical and research needs in the acutephase and the long return to normality. Child Adolesc. Psychiatryand Ment. Health.

[18] Frühauf A., Schnitzer M., Schobersberger W., Weiss G., Kopp M. (2020). Jogging, nordic walking and going for a walk-inter-disciplinary recommendations to keep people physically active in times of the COVİD-19 lockdown in Tyrol, Austria. Curr. Issues Sport Sci. (CISS).

[19] George D., Mallery M. (2010). SPSS For Windows Step by Step: A Simple Guide and Reference.

[20] Gerber M., Brand S., Elliot C., Holsboer-Trachsler E., Pühse U. (2014). Aerobic exercise, ball sports, dancing, and weight lifting as moderators of the relationship between stress and depressive symptoms: An exploratory cross-sectional study with swiss university students. Percept. Mot. Ski..

[21] Gilbert P., Irons C. (2009). Shame, self-criticism, and self-compassion in adolescence. Adolesc. Emot. Dev. Emerg. Depress. Disord..

[22] Gilbert P., McEwan K., Catarino F., Baião R., Palmeira L. (2014). Fears of happiness and compassion in relationship with depression, alexithymia, and attachment security in a depressed sample. Br. J. Clin. Psychol..

[23] Gilman R., Huebner S. (2003). A review of life satisfaction research with children and adolescents. Sch. Psychol. Q..

[24] Gökçen A. (2019). Investigation of the Effects of Dance/Movement Therapy on Psychotic Status and Functional Remission in Individuals with Schizophrenia: Randomized Controlled Study. Unpublished. Doctoral Thesis.

[25] Greenberg M.T., Weissberg R.P., O’Brien M.U., Zins J.E., Fredericks L., Resnik H., Elias M.J. (2023). Enhancing school-based prevention and youth development through coordinated social, emotional, and academic learning. Am. Psychol..

[26] Guisado E.U., Sanchez J.S. (2019). Effects of zumbaand aquagym on bone mass in inactive middle aged. Medicina.

[27] Han J.H., Sa H.J. (2023). Health and happiness of older Korean women participating in dance activities. Heliyon.

[28] Hanna J.L. (2014). Dancing to Learn: The Brain’s Cognition, Emotion, and Movement.

[29] Hewston P., Kennedy C.C., Borhan S., Merom D., Santaguida P., Ioannidis G., Marr S., Santesso N., Thabane L., Bray S. (2021). Effects of dance on cognitive function in older adults: A systematic review and meta-analysis. Age Ageing.

[30] Ji H. (2024). Using dance-based interventions to overcome anxiety and stress in China. Curr. Psychol..

[31] Joshanloo M. (2013). The influence of fear of happiness beliefs on responses to the satisfaction with life scale. Personal. Individ. Differ..

[32] Karkou V., Sanderson P. (2006). Arts Therapies: A Research-Based Map of the Field.

[33] Koch S., Kunz T., Lykou S., Cruz R. (2014). Effects of dance movement therapy and dance on health-related psychological outcomes: A meta-analysis. Arts Psychother..

[34] Lim Y., Song J. (2019). Art Therapy for Stress Reduction in College Students: A Randomized Controlled Study. J. Korean Med. Sci..

[35] Liu X., Shen P.L., Tsai Y.S. (2021). Dance intervention effects on physical function in healthy older adults: A systematic review and meta-analysis. Aging Clin. Exp. Res..

[36] Lobo Y.B., Winsler A. (2006). The effects of a creative dance and movement program on the social competence of head start preschoolers. Soc. Dev..

[37] Papacharisis V., Goudas M., Danish S.J., Theodorakis Y. (2005). The effectiveness of teaching a life skills program in a sport context. J. Appl. Sport Psychol..

[38] Quiroga Murcia C., Kreutz G., Clift S., Bongard S. (2010). Shall we dance? An exploration of the perceived benefits of dancing on well-being. Arts Health.

[39] Ruseski J.E., Humphreys B.R., Hallmann K., Breuer C. (2011). Family structure, time constraints, and sport participation. Eur. Rev. Aging Phys. Act..

[40] Saulle R., De Sario M., Bena A., Capra P., Culasso M., Davoli M., De Lorenzo A., Lattke L.S., Marra M., Mitrova Z. (2022). School closures and mental health, wellbeing and health behaviours among children and adolescents during the second COVID-19 wave: A systematic review of the literature. Epidemiol. Prev..

[41] Silva R.M.F., Fonseca Terra L., Fernandes M.d.S.V., Noll P.R.E.S., Abreu L.C.d., Noll M. (2022). Barriers to physical activity among full-time students: A case studyduring the COVID-19 pandemic. Sustainability.

[42] Suldo S.M., Huebner E.S. (2006). Is extremely high life satisfaction during adolescence advantageous?. Soc. Indic. Res..

[43] Terzi G. (2005). Psychological Hardiness Model Relating to Subjective Well Being. Unpublished. Doctoral Thesis.

[44] Tunçgenç B., Greig E.J., Cohen E. (2024). Benefits of an on-line group dance program for adolescents’ social bonding and wellbeing. J. Adolesc..

[45] Yumuşak M. (2019). The Levels of Smart Phone Addiction, Sleep Quality and Satisfaction with Life in University Students. Master’s Thesis.

[46] Zolopa C., Burack J.A., O’Connor R.M., Corran C., Lai J., Bomfim E., DeGrace S., Dumont J., Larney S., Wendt D.C. (2022). Changes in youth mental health, psychological wellbeing, and substance use during the COVID-19 pandemic: A rapid review. Adolesc. Res. Rev..

[47] Zullig K.J., Valois R.F., Huebner E.S., Drane J.W. (2005). Associations among family structure, demographics and adolescent perceived life satisfaction. J. Child Fam. Stud..

[48] Zygmont A., Doliński W., Zawadzka D., Pezdek K. (2023). Uplifted by Dancing Community: From Physical Activity to Well-Being. Int. J. Environ. Res. Public Health.

